# Future Climate Significantly Alters Fungal Plant Pathogen Dynamics during the Early Phase of Wheat Litter Decomposition

**DOI:** 10.3390/microorganisms8060908

**Published:** 2020-06-16

**Authors:** Sara Fareed Mohamed Wahdan, Shakhawat Hossen, Benjawan Tanunchai, Martin Schädler, François Buscot, Witoon Purahong

**Affiliations:** 1 Department of Soil Ecology, UFZ-Helmholtz Centre for Environmental Research, Theodor-Lieser-Str. 4, 06120 Halle (Saale), Germany; shakhawat.hossen@ufz.de (S.H.); tanunchai.benjawan@ufz.de (B.T.); francois.buscot@ufz.de (F.B.); 2 Department of Botany, Faculty of Science, Suez Canal University, 41522 Ismailia, Egypt; 3 Friedrich-Schiller-Universität Jena, Institute of Ecology and Evolution, Dornburger Str. 159, 07743 Jena, Germany; 4 Department of Community Ecology, UFZ-Helmholtz Centre for Environmental Research, Theodor-Lieser-Str. 4, 06120 Halle (Saale), Germany; martin.schaedler@ufz.de; 5 German Centre for Integrative Biodiversity Research (iDiv) Halle-Jena-Leipzig, Deutscher Platz 5e, 04103 Leipzig, Germany

**Keywords:** wheat straw decomposition, litter decomposition, climate change, mycobiome, MiSeq Illumina sequencing, fungal ITS2, *GCEF*

## Abstract

Returning wheat residues to the soil is a common practice in modern agricultural systems and is considered to be a sustainable practice. However, the negative contribution of these residues in the form of “residue-borne pathogens” is recognized. Here, we aimed to investigate the structure and ecological functions of fungal communities colonizing wheat residues during the early phase of decomposition in a conventional farming system. The experiment was conducted under both ambient conditions and a future climate scenario expected in 50–70 years from now. Using MiSeq Illumina sequencing of the fungal internal transcribed spacer 2 (ITS2), we found that plant pathogenic fungi dominated (~87% of the total sequences) within the wheat residue mycobiome. Destructive wheat fungal pathogens such as *Fusarium graminearum*, *Fusarium tricinctum,* and *Zymoseptoria tritci* were detected under ambient and future climates. Moreover, future climate enhanced the appearance of new plant pathogenic fungi in the plant residues. Our results based on the bromodeoxyuridine (BrdU) immunocapture technique demonstrated that almost all detected pathogens are active at the early stage of decomposition under both climate scenarios. In addition, future climate significantly changed both the richness patterns and the community dynamics of the total, plant pathogenic and saprotrophic fungi in wheat residues as compared with the current ambient climate. We conclude that the return of wheat residues can increase the pathogen load, and therefore have negative consequences for wheat production in the future.

## 1. Introduction

Plant residues play important roles in nutrient dynamics and soil fertility in different types of agricultural ecosystems [1]. Consequently, the return of such residues to the soil is used to improve crop yields [2] not only by improving soil structure but also by increasing organic matter content, reducing evaporation, and helping to fix carbon dioxide in the soil [3]. Incorporation of plant residue into the soil provides an opportunity to limit soil organic matter depletion resulting from cultivation, and it also represents a valuable source of nutrients such as nitrogen, thereby contributing to protection of farmland eco-environments [4,5]. It has been reported that incorporating straw into the soil significantly increases wheat yield by an average of 58% as compared with straw removal [6]. Moreover, soil fertility has been found to be improved after straw incorporation by increasing available nitrogen, phosphorus, and potassium by more than 15%, while organic carbon increased to 8–22% [6].

Plant residues are not considered to be a static habitat [7] as they are a transient substrate between plant and soil. The initial microbiome that colonizes plant residues is inherited from living plants and depends on their species identity [8]. Since plant residues decay in soils within one or more cropping seasons, it is highly likely that there will be further colonization by autochtonous soil saprotrophs since these grow abundantly on decaying organic matter. Hence, over time, we can expect strong microbial community dynamics and diverse ecological functions in plant residues. Whilst some of these early microbial populations in residues prevent diseases or enhance plant growth, others cause different types of plant disease [9]. For example, the wheat pathogens *Zymoseptoria tritici* [10] and *Oculimacula yallundae* [11], colonize wheat residues and are able to infect the subsequent crop if the residues are left in the field after harvest. Hence, residue return can be considered to be a causative agent for plant diseases, by providing pathogen inoculum and suitable conditions for pathogen growth, propagation, and accumulation, which then results in epidemic diseases [2,7]. However, these complex microbial communities inhabiting plant residues have remained largely uncharacterized [12]. Recent studies, using next generation sequencing (NGS), have greatly improved our understanding of the richness and composition of mycobiomes associated with plant residues [9,13,14,15,16,17,18,19], however, the approach has not yet been applied to investigate the fungal pathogen gains and their dynamics in plant residues.

Current climate change is associated with increases in temperatures and decreases in precipitation patterns and seasonal and perennial snow and ice extent [20], which can have an effect on pathogens [21] by altering their seasonal phenology (e.g., life cycle stages and rates of the development of the pathogen) and their population dynamics (e.g., over-wintering, over-summering, survival, and changes in abundance) [22,23,24,25,26,27]. Despite phytopathogenic microbes representing a major threat to agriculture and food security [28], the effect of climate change on the diversity and dynamics of wheat residue-inhabiting mycobiomes, and therein of fungal pathogens, are not yet known. In this context, we used NGS (Illumina MiSeq sequencing) of the fungal internal transcribed spacer 2 (ITS2) region to study the mycobiome associated with wheat residues in soil at an early decomposition stage. The experiment was performed at the *Global Change Experimental Facility*, an experimental field infrastructure to compare ecosystem effects under actual climate in central Germany and under a scenario projected for a period of 50–70 years [29].

The aims of this study were the following: (i) To investigate the wheat residue mycobiome during the early phase of decomposition (0, 30, and 60 days) under current and future climate conditions and (ii) to quantify the richness and proportion of fungal pathogens within these mycobiomes. We hypothesized the following: (i) diverse taxonomic and functional groups of fungi are present in wheat residues; (ii) saprotrophs followed by plant pathogens dominate the mycobiome, especially at 60 days after the beginning of decomposition; (iii) saprotroph richness and relative abundances increase over time while those of plant pathogens decrease; (iv) there are differences in the initial communities colonizing wheat straw under the ambient climate as compared with the simulated future climate; and (v) the future climate has a significant differential effect on pathogen dynamics in plant residues in agricultural ecosystems.

## 2. Materials and Methods

### 2.1. Study Site, Experimental Setup, and Design

The study was conducted within the *Global Change Experimental Facility* (*GCEF*) at the field research station of the Helmholtz Centre for Environmental Research in Bad Lauchstädt, Saxony-Anhalt, Germany (51°22′60 N, 11°50′60 E, 118 m a.s.l.). The area is characterized by a subcontinental climate with a mean temperature of 8.9 °C and a low mean annual rainfall of 498 mm (long-term mean 1896–2013) resp. 9.8 °C and 516 mm (1995–2014). The time period of our experiment (2018) has been recorded as the warmest year in Germany since 1881.

The *GCEF* is a large field experiment for the investigation of the consequences of climate change on ecosystem processes under different land use types [29]. Half of the field plots are subjected to a future climate scenario based on several models (COSMO-CLM [30], REMO [31], and RCAO [32]) of climate change predicting the climate in Central Germany for the years between 2070 and 2100. We first used 12 climate simulations based on the tree models. These different simulations produce a variety of projections for future climate. Therefore, we used the mean values of projections of climate change across the different climate simulations [29]. The resulting scenario included manipulation of both precipitation and temperature. For this, future climate plots (Figure S1) are equipped with mobile shelters and side panels, as well as an irrigation system, the roofs are controlled by a rain sensor. As a result of continuous adjustment of irrigation or roof closing, precipitation is reduced by ~20% in summer months and increased by ~10% in spring and autumn. To simulate the increase in temperature with asymmetry between daytime and nighttime warming, we used the standard method “passive nighttime warming”. The shelters and panels automatically close from sundown to sunrise to increase the mean daily temperature by ~0.55 °C. The resulting changes in climate conditions before and during the study period are shown in Figure S2. Ambient climate plots (Figure S1) are equipped with the same steel constructions (but without shelters, panels, and irrigation system) to mimic possible microclimatic effects of the experimental setup [29]. Five land use types are involved in factorial combination with the two climate scenarios, on split plots under the roofs, and the resulting blocks are replicated five times. The land use treatments consist of three grasslands (conventional mown, organic mown and organic grazed) and two farming scenarios (conventional and organic). The wheat straw decomposition experiment was performed on the conventional farming plots under both ambient (five plots) and future climate (five plots) conditions. The conventional farming plots are characterized by a typical regional crop rotation (including winter rape, winter wheat, and winter barley) with tillage and application of mineral fertilizers and pesticides. Details of the management are given in [29].

### 2.2. Litterbag Preparation, Field Incorporation, and Sampling

The straw left over (10 cm aboveground) from harvested winter wheat (*Triticum aestivum* L.) was sampled from each *GCEF* field plot and placed in sterile plastic bags before being transported to the laboratory on ice. The wheat straw from each plot was oven-dried at 25 °C for 3 days to normalize the moisture content, and then 10 g was enclosed in a litter bag (20 × 15 cm, 5 mm mesh size). Three litter bags were returned back to each field plot (five ambient conventional farming plots and five future conventional farming plots) in midAugust, 2018. We evaluated the effects of future climate four years after the start of climate manipulation (The manipulation of temperature and precipitation started in April and July 2014, respectively). To simulate the natural field conditions, the litterbags were placed on the soil surface at the beginning of the experiment. The first sampling of litterbags occurred at the onset of the experiment (0 days). After the process of tillage in September, the litterbags were buried at the same original location in the plots. Two further samples were collected, the first 30 days and the second 60 days after field incorporation (Figure S1).

### 2.3. DNA Extraction, PCR, and Illumina Miseq Sequencing

Wheat straw from each bag was homogenized with the aid of liquid nitrogen and 150 mg of the ground material was used for DNA extraction using a DNeasy PowerSoil kit (Qiagen, Valencia, CA, USA) according to the manufacturer’s instructions. For identification of the fungal taxa, the internal transcribed spacer 2 (ITS2) region of the rRNA was amplified using the fungal-specific forward primer fITS7 (5′-GTGARTCATCGAATCTTTG-3′) [33] and the reverse primer ITS4 (5′-TCCTCCGCTTATTGATATGC-3′) [34]. To generate the fungal amplicon library, DNA amplification was conducted in a two-step process. During the first PCR, the forward primer was constructed with the Illumina i5 sequencing primer (5′-TCGTCGGCAGCGTCAGATGTG TATAAGA GACAG-3′) and the fITS7 primer. The reverse primer was constructed with the Illumina i7 sequencing primer (5′-GTCTCGTGGG CTCGGAGATGTGTATAAGAGACAG-3′) and the ITS4 primer. Amplifications were performed in 25 µL reactions with a Qiagen HotStar Hi Fidelity Polymerase Kit (Qiagen Inc., Valencia, CA, USA), 1 µL of each 5 µM primer, and 1 µL of template. Reactions were performed on ABI Veriti thermocyclers (Applied Biosytems, Carlsbad, CA, USA). The PCR thermal profile was 95 °C for 5 min, then 35 cycles of 94 °C for 15 s, 54 °C for 60 s, 72 °C for 1 min, followed by one cycle of 72 °C for 10 min and 4 °C hold. Products from the first stage amplification were added to a second PCR based on qualitatively determined concentrations. During the second PCR, dual indexes were attached using the Nextera XT Index Kit with the same amplification conditions as the first stage except with 10 cycles rather than 35. Amplification products were visualized with eGels (Life Technologies, Grand Island, NY, USA). Then, products were pooled in equimolar quantities and each pool was size selected in two rounds using Agencourt AMPure XP (BeckmanCoulter, Indianapolis, IN, USA) in a 0.75 ratio for both rounds. Then, size selected pools were quantified using the Quibit 2.0 fluorometer (Life Technologies). Sequencing was performed using MiSeq (Illumina, Inc. San Diego, CA, USA) 2 × 300 bp paired-end strategy.

### 2.4. Detection of the Metabolically Active Mycobiome Colonizing Wheat Residues

To characterize metabolically active fungi involved in wheat straw decomposition, we used the bromodeoxyuridine (BrdU) immunocapture technique [35]. Briefly, a thymidine analog, bromodeoxyuridine (BrdU), was added to the wheat straw residues (at initial and early decomposition stages) and incubated for two days in the field. DNA was extracted, and the newly synthesized BrdU-labeled DNA was isolated by immunocapture using specific anti-BrdU antibodies as previously described [35]. PCR and Illumina sequencing were performed as described above.

### 2.5. Sequence Processing

Merging of the demultiplexed raw reads pair-end data was achieved using the simple Bayesian algorithm with a threshold of 0.6 and a minimum overlap of 20 nucleotides as implemented in PANDAseq [36]. All assembled reads were high quality filtered (minimum sequence length 120 bp, maximum sequence length 580 bp, minimum average Phred score of 25, and maximum length of 20 homopolymers in the sequence and without ambiguous nucleotides). Potential chimeras were removed using UCHIME [37] as implemented in MOTHUR [38]. High-quality reads were clustered into operational taxonomic units (OTUs) using cd-hit-est 4.6.2 [39] at a threshold of 97% pairwise similarity. To assess taxonomic affiliations, the reads were assigned to the UNITE v. 7 sequence database [40] using the Bayesian classifier as implemented in MOTHUR [38]. The sequences that could not be classified at the kingdom level were removed from the dataset. Singletons and doubletons, which could have originated from sequencing errors, were also removed. The datasets were normalized to the minimum number of sequencing reads per sample using the function of ”rrarefy” from the vegan package [41] in the R environment version 3.6.1. [42]. Putative ecological functions were assigned to the fungal OTUs using the annotation tool, FUNGuild [43]. Fungal genera that only classified to a functional guild with ”probable” or ”highly probable” confidence were considered for further statistical analysis. Important functions were added based on a literature survey. All OTUs assigned as plant pathogenic fungi corresponded to the species hypothesis ”SH” in the UNITE [44] database. Then, SH created using ≥99% similarity threshold was modified according to the current name in the Index Fungorum database. The table of main species along with the full name according to Index Fungorum are included in Table S1. The raw sequences of fungal datasets have been deposited in the National Center for Biotechnology Information (NCBI) Sequence Read Archive (SRA) under BioProject accession number: PRJNA624995 (https://www.ncbi.nlm.nih.gov/sra/?term=PRJNA624995).

### 2.6. Statistical Analysis

Statistical analyses were performed using the R software [42] and PAST program v. 2.17c [45]. All the analyses were conducted based on five independent replicates of the field experiment (*n* = 5). The observed richness of OTUs was calculated for each sample using PAST. The sample-based rarefaction curves indicated saturation of fungal diversity at the analyzed sequencing depth for most samples, and thus we used the observed OTU richness directly as a proxy for fungal diversity. Non-metric multidimensional scaling (NMDS) using the Jaccard dissimilarity measure was carried out to describe fungal community structures in relation to sampling times and climate treatments using the vegan package [41]. Permutational multivariate analysis of variance (NPMANOVA) based on Jaccard distance was performed to test dynamics of fungal communities at each time point (0, 30, and 60 days) subjected to ambient and future climate treatments. As relative abundances obtained by NGS could not be used quantitatively, we mostly used presence/absence data for multivariate statistics. Two-way analyses of variance (ANOVA) incorporating the Jarque-Bera (JB) test for normality were performed to test the impact of climate change and sampling times on the OTU richness of the overall community and on the richness of OTUs assigned as saprotrophs and plant pathogens. A *t*-test was applied to evaluate differences in relative abundance of dominant fungal genera at 0 days under ambient and future climate treatments. The hierarchical structure of taxonomic classification of the detected fungal OTUs is presented in a heat tree (based on presence/absence data) created by the metacoder package [46] in the R software.

## 3. Results

### 3.1. Processing of the Sequences

**Figure 1:**
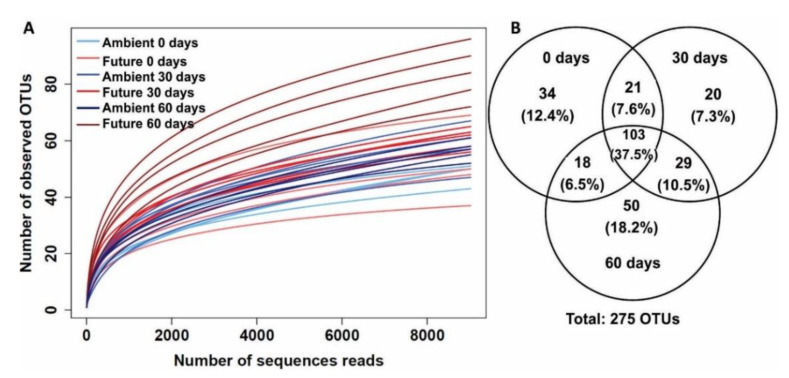
Overview of retrieved fungal operational taxonomic units (OTUs). (**A**) Rarefaction curve of 30 samples at an OTU threshold of 97% sequence similarity; (**B**) Venn diagram showing the overlap and distribution of fungal OTUs colonizing wheat straw at three sampling time points (0, 30, and 60 days).

After removal of singletons and doubletons, a total of 585,217 high quality sequences were obtained among the 30 samples with an average of 19,507 sequences per sample. For normalization, the dataset was rarefied to 9020 sequence reads per sample (Figure 1A).

At 97% similarity threshold, the number of fungal OTUs colonizing wheat straw was 275 (Figure 1B). The retrieved fungal OTUs belonged to Ascomycota (252 OTUs), Basidiomycota (20 OTUs), Chytridiomycota (one OTU), and two OTUs of unclassified fungi. By analyzing the OTU distribution at the three time points, we found that 12.4%, 7.3%, and 18.2% of OTUs were unique to samples collected at 0, 30, and 60 days, respectively (Figure 1B). At 0 days, 24.4% and 33.5% were unique to ambient and future climate conditions, respectively, whereas only 42% of OTUs were shared between both climate treatments (Figure S3).

### 3.2. Climate-Dependent Significant Changes in Composition, Taxonomy, and Richness of the Fungal Community during Early Stages of Wheat Straw Decomposition

**Figure 2:**
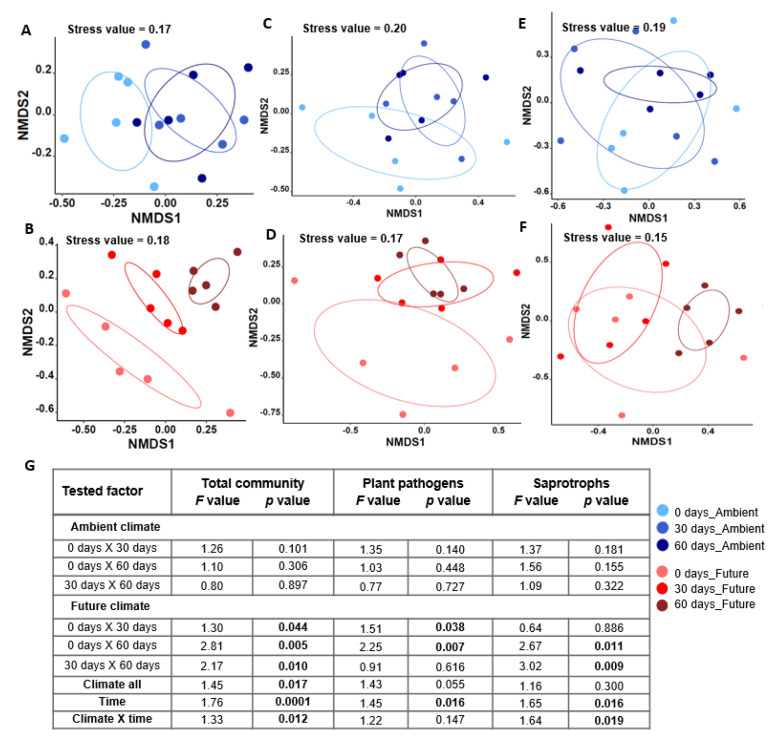
Non-metric multidimensional scaling (NMDS) ordination diagrams of the overall fungal community colonizing wheat straw residues in (**A**) ambient and (**B**) future climate treatments; The plant pathogenic community in (**C**) ambient and (**D**) future climate treatments; The saprotrophic community in (**E**) ambient and (**F**) future climate treatments. NMDS based on Jaccard dissimilarities was used to determine the compositional variation, enclosed areas in the NMDS plot are 95% confidence ellipses, stress (data distortion) values representing the difference between distances in the reduced dimension as compared to the complete multidimensional space (a commonly accepted stress value = 0.20); (**G**) Results of the permutational multivariate analysis of variance (NPMANOVA) test analyzing the effects of decomposition time (sampling date), climate changes, and the interaction between the two factors on the dynamics of the overall, plant pathogenic and saprotrophic fungi colonizing wheat straw residues. Significant results (*p* < 0.05) from NPMANOVA are indicated in bold.

Total fungal communities displayed different dynamic patterns under the ambient and future climate treatments during the early stages of residue decomposition. In the ambient climate treatment, the NMDS analysis showed that fungal communities overlapped at the three sampling times (Figure 2A). This result was confirmed by NPMANOVA (Figure 2G), showing that fungal communities did not differ significantly (*p* > 0.05) among 0, 30, and 60 days. In contrast, under the future climate treatment, the fungal communities at each sampling time were distinct from each other (Figure 2B). NPMANOVA (Figure 2G) also indicated that fungal communities colonizing wheat straw were significantly different among all sampling times.

**Figure 3:**
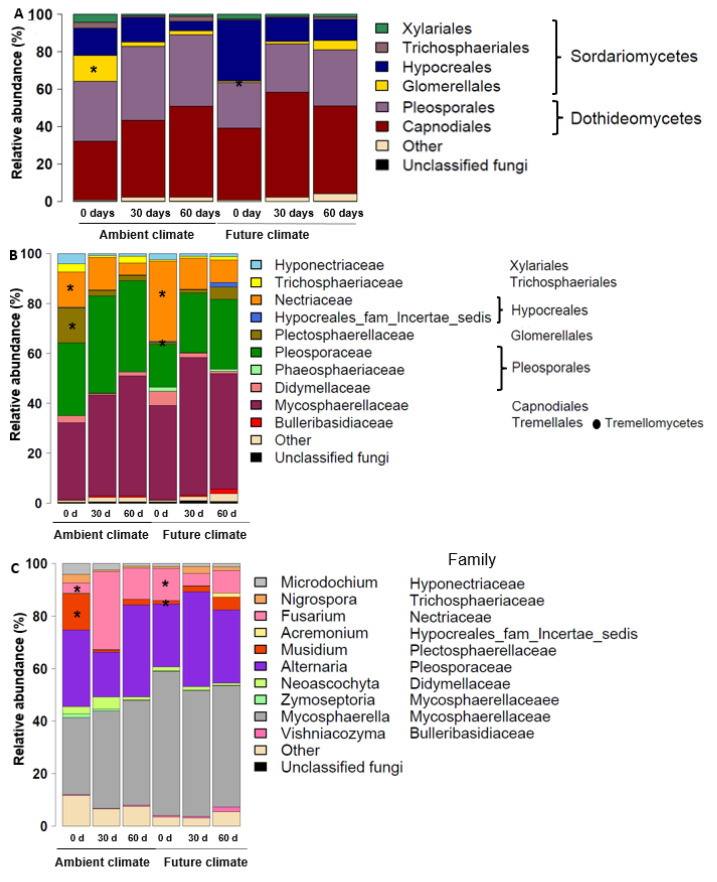
Relative abundance of the dominant fungal taxa at (**A**) order, (**B**) family, and (**C**) genus level at different sampling times (0, 30, and 60 days) under ambient and future climate treatments. Asterisks indicate taxa that differ significantly under ambient and future conditions at 0 days (*t*-test, *p* < 0.05).

**Figure 4:**
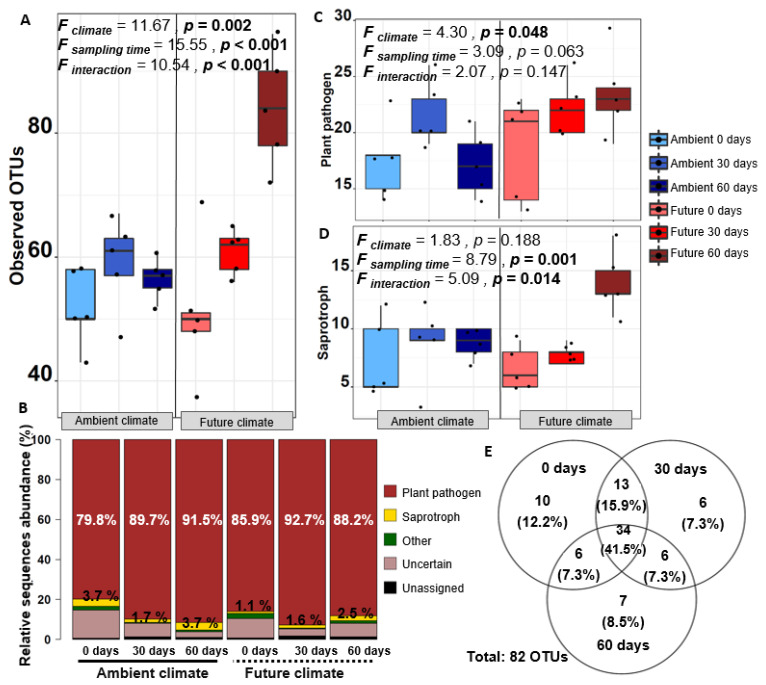
(**A**) Box plots showing observed OTUs at each time point (0, 30, and 60 days) under ambient and future climate treatments, supported by the results of a two-way ANOVA; (**B**) Stacked bar chart showing the relative abundance of predicted fungal guilds using the FUNGuild annotation tool; Box plots showing observed richness of OTUs classed as (**C**) plant pathogens and (**D**) saprotrophs; (**E**) Venn diagram showing the overlap and distribution of fungal OTUs classed as plant pathogens, which colonize wheat straw at the three sampling time points (0, 30, and 60 days).

The analysis of the taxonomic composition of the wheat residue mycobiome revealed that Sordariomycetes and Dothideomycetes were the dominant classes at all time points under ambient and future climate treatments (Figure 3A,B). At genus level, Mycosphaerella (~42% of all sequences), Alternaria (~28% of all sequences) and Fusarium (~12% of all sequences) dominated the community (Figure 3C). By investigating the effect of climate treatment on the dominant genera at the beginning of the experiment (0 days), we found that some of the genera were highly influenced by specific climate treatment. Relative abundance of *Fusarium* spp. significantly increased (*t* = 1.4, *p* < 0.05) under the future climate treatment as compared with ambient climate treatment, while relative abundance of *Musidium* spp. significantly reduced (*t* = −2.5, *p* < 0.05). In terms of observed fungal OTU richness at different time points under both climate treatments (Figure 4A), two-way ANOVA indicated that both climate and sampling time significantly affected the fungal community (Figure 4A) especially under the future climate treatment, as OTU richness significantly increased with time.

### 3.3. Plant Pathogens Dominate the Fungal Guilds

The fungal community colonizing wheat straw was assessed in terms of fungal guilds and trophic modes using FUNGuild (Figure 4B). Plant pathogenic fungi dominated the fungal communities (relative abundance 79.8–92.7%) at all time points and under both climate treatments, while saprotrophs were present at relatively low abundance (1.1–3.7%) among all treatments (Figure 4B). In addition, plant pathogens were represented by a higher number of OTUs than saprotrophs (Figure 4C,D). In total, 82 OTUs classed as plant pathogenic fungi colonized the wheat straw residues at the three sampling time points (Figure 4E). The analysis revealed a significant effect of climate treatment only on plant pathogenic fungi (*F* = 4.30, *p* = 0.04). A noticeable increase in OTU richness related to plant pathogens was observed under the future climate treatment (Figure 4C). On the other hand, only saprotrophs responded significantly to the sampling time (*F* = 8.79, *p* = 0.001) and interaction between climate and sampling time (*F* = 5.09, *p* = 0.014), especially under the future climate treatment, which was characterized by an increase in OTUs related to saprotrophs (Figure 4D) over time.

### 3.4. Strong Succession Pattern of Pathogenic and Saprotrophic Fungi Colonizing Wheat Straw Residues over Time under the Future Climate Treatment but Not under Ambient Climate

Ambient climate had no significant effect on the dynamics of either plant pathogenic (Figure 2C,G) or saprotrophic fungi (Figure 2E,G) colonizing wheat straw at the different sampling times (0, 30, and 60 days). In contrast, the future climate was associated with a significant shift in the plant pathogenic community colonizing wheat straw at 30 (*F* = 1.51, *p* = 0.038) and 60 days (*F* = 2.25, *p* = 0.007) of field incorporation as compared with the community at 0 days (Figure 2D,G). A compositional shift of saprotrophic communities was detected at 60 days as compared with the community at 0 days (*F* = 2.67, *p* = 0.011). Similarly, saprotrophic communities at 30 and 60 days differed significantly (*F* = 3.02, *p* = 0.009) only in the case of the future climate treatment (Figure 2F,G).

### 3.5. Future Climate Affects the Taxonomic Distribution of Plant Pathogenic Fungi

**Figure 5:**
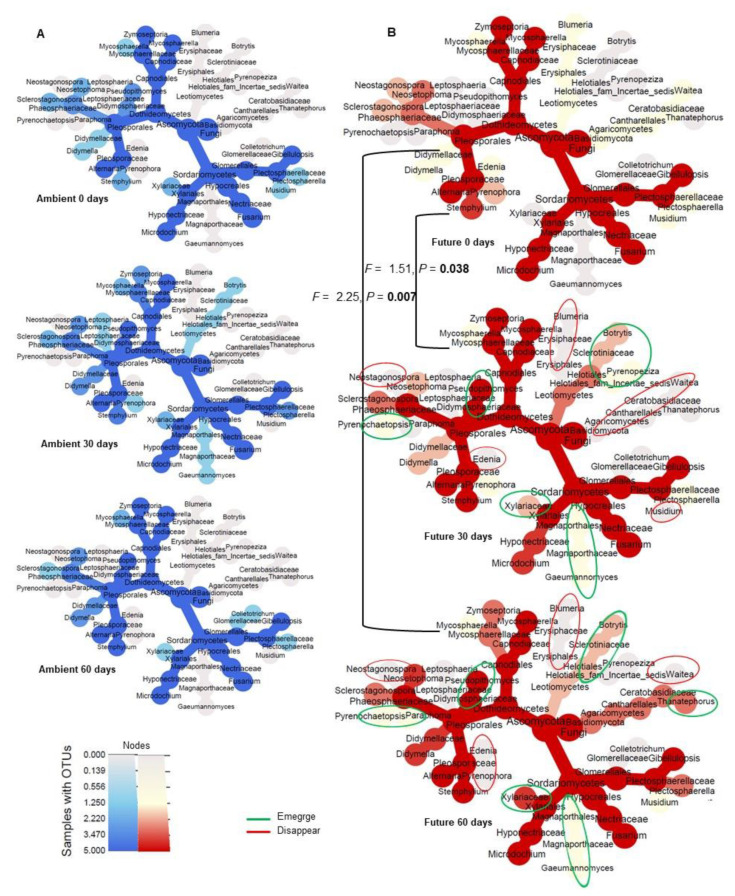
Microbial composition of the plant pathogenic fungal community colonizing wheat straw residues. Heat tree showing the fungal community composition at three sampling time points (0, 30, and 60 days) under ambient (**A**) and future (**B**) climate treatments. Colors represent the occurrences of OTUs. Gain of new taxa colonizing wheat straw is indicated by green circles while loss of taxa is indicated by red circles. The NPMANOVA test (Figure 2G) confirmed significant dynamics of the pathogenic mycobiome under the future climate treatment.

We analyzed the taxonomic distribution of fungal OTUs classed as plant pathogens after this was revealed to be the dominant functional group (Figure 5A,B). We observed that some genera were specific to each climate treatment. For example, Leptosphaeria and Colletotrichum were detected only in the ambient climate plots, while *Blumeria*, *Paraphoma*, *Pyrenopeziza*, *Waitea*, *Thanatephorus*, Edenia, Pyrenochaetopsis, and Neostagonospora were detected only in the future climate plots.

The dynamics of the plant pathogenic community at the three sampling times were clearly detectable only under the future climate treatment (Figure 5B). Some taxa that initially colonized the wheat straw residues (0 days) totally disappeared at 30 or 60 days (Blumeria (Erysiphaceae), Edenia (Pleosporaceae), Neostagonospora (Phaeosphaeriaceae), and Waitea (Ceratobasidiaceae)). New colonizing taxa emerged at both 30 and 60 days, including Gaeumannomyces (Magnaporthaceae), Botrytis (Sclerotiniaceae), Pseudopithomyces (Didymosphaeriaceae), and Pyrenochaetopsis (Phaeosphaeriaceae). Thanatephorus (Ceratobasidiaceae) was detected at the last time point (60 days). By increasing the field incubation time from 0–60 days, some taxa exhibited higher occurrence, these included Didymella (Didymellaceae) and Sclerostagonospora (Phaeosphaeriaceae), whereas others exhibited lower occurrence, these included Zymoseptoria (Mycosphaerellaceae).

### 3.6. Future Climate Could Encourage New Pathogenic Fungal Species to Colonize Wheat Straw Residues

**Figure 6:**
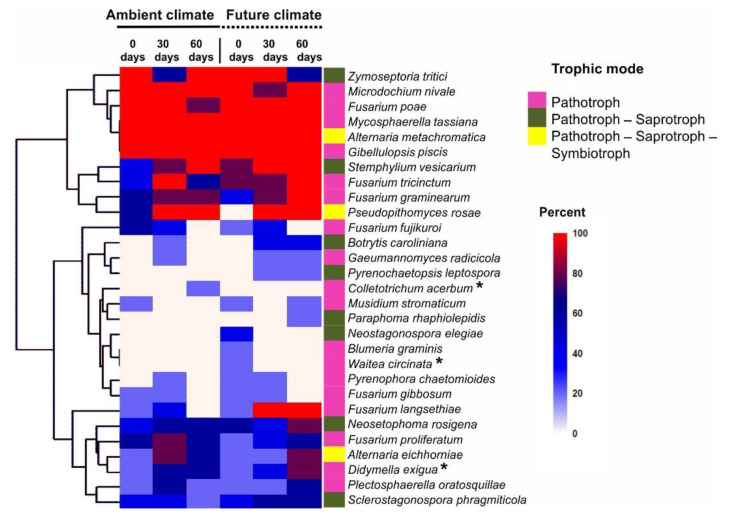
Heat map of all OTUs classed as pathotrophic fungi found to colonize wheat straw residues. The OTUs corresponded to the ”species hypothesis” in the UNITE database using ≥99% similarity threshold. Three main clusters of species were found based on the occurrence of OTUs at different sampling times of wheat straw under ambient and future climate treatments. Non-active pathogens are indicated by asterisks.

According to the species hypothesis of the UNITE database, 29 fungal species were retrieved and constituted three occurrence clusters in relation to the treatments (Figure 6). The first cluster comprised all species with high occurrence at almost all time points under both ambient and future climate treatments, these included *Mycosphaerella tassiana*, *Alternaria metachromatica*, *Fusarium poae*, *F. tricinctum*, *F. graminearum*, *Microdochium nivale* and *Gibellulopsis piscis*. The second cluster contained most species with relatively low occurrence at almost all sampling time points, but present in both ambient and future climate treatments, these included *F. proliferatum,* *Plectosphaerella oratosquillae,* *Sclerostagonospora phragmiticola* *Neosetophoma rosigena*, and *Didymella exigua*. The exception was for *Fusarium langsethiae* that shows high occurrence at 30 and 60 days under the future climate scenario. The third cluster consisted of species with the lowest occurrence or species detected under a specific climate treatment. Unique species were colonizing the plant residues under the future climate treatment at 0 days (*Blumeria graminis*, *Waitea circinate,* and *Neostagonospora elegiae*), at 0 and 30 days (*Pyrenochaetopsis leptospora*), and at 60 days (*Paraphoma rhaphiolepidis*). The results based on BrdU-labeled DNA showed that almost all the detected pathogens (26/29 species) were metabolically active. Exceptions were *Waitea circinata*, *Didymella exigua*, and *Colletotrichum acerbum*.

## 4. Discussion

In this work, we quantified the richness of functional groups of fungi and their dynamics in wheat residues under ambient and future climate conditions. The future climate scenario is characterized by a reduction of precipitation by ~20% and increasing in soil temperature by ~1.5 °C during the period of our experiment (Figure S1) as comparing with the current climate conditions. The future climate scenario significantly changes the richness patterns and community dynamics of total, saprotroph, and plant pathogenic fungi. Importantly, we found a consistent pattern under both climate treatments, namely that fungal pathogens dominated the mycobiome and almost all of them were active. However, our results also show that the future climate treatment brings new plant pathogens into the system (as compared with ambient climate treatment) and significantly affects the pathogen community dynamics during the first 60 days of decomposition. These changes could affect subsequent wheat production if the residues are returned to the soil.

### 4.1. The Hyper Dominant Pattern of Fungi Inhabiting Wheat Residue Remains until 60 Days

Using the internal transcribed spacer 2 (ITS2) metabarcoding of fungi, our study on the dynamics of the mycobiome reveals a hyper dominant pattern of Sordariomycetes and Dothideomycetes occurring consistently under both climate treatments up to 60 days of decomposition. Comparing the two classes, Dothideomycetes are highly dominant (covering ~60% and 80% of sequence relative abundance at 0 and 60 days, respectively). The most frequently detected genera were Mycosphaerella and Alternaria, which are clearly resistant to the climate change scenario considered. Although both are potential plant pathogens [47], they are frequently detected on wheat crop residues during their saprophytic life stage. Consistent with other studies [8], Ascomycota is the dominant fungal phylum in wheat residues and is represented by Alternaria, Cladosporium, and Phaeosphaeria. Ascomycota includes different soil-borne fungal plant pathogens, as well as nonpathogenic genera and their abundance is higher in low productivity land [48].

### 4.2. Robustness of Initial Fungal Wheat Residue Inhabitants to Mechanical Disturbance and Climate Change

Unexpectedly, we found that potentially active phytopathogens dominated the mycobiome in plant residues (relative abundance on average 87% among all fungal microbiota for each climate at the initial time point, 0 days). Among them, *Zymoseptoria tritci*, *Microdochium nivalis*, *Gibellulopsis piscis*, *Mycosphaerella tassinia*, *Stemphylium vesicarium*, *Fusarium poae*, *F. graminearum*, *F. tricinctum*, and *F. langsethiae* were detected. We confirmed that this zymogenic community has been inherited from the wheat plant, as reported previously [8,49]. Although wheat residues were buried in the soil after tillage (before 30- and 60-day sampling), the colonizing pathogens continued to be detected in large numbers throughout the first 60 days of straw decomposition, in both ambient and future climate treatments. The reason is that many pathogens, either necrotrophs (e.g., *Zymoseptoria tritci*, *Stemphylium vesicarium*, *Microdochium nivale*, etc.), or biotrophs (e.g., *Blumeria graminis*) can use plant residues as shelter [50]. They can over-summer or over-winter in the form of dormant structures within plant debris [47,50]. Pathogens induce changes in plant tissues, which can themselves modify the fungal communities inhabiting the residues and this general phenomenon can demonstrate the impact of pathogens on the fungal communities observed in both ambient and future climate treatments [51].

The viability of plant pathogens for 60 days in wheat residues is sufficient for the seedling blight pathogens *F. culmorum*, *F. graminearum*, *F. poae,* and *Microdochium nivalis* to infect the subsequent wheat crop at the seedling stage [52,53]. Moreover, *Fusarium graminearum* and *Fusarium culmorum* cause devastating diseases at different life stages of wheat, including foot and root rot, and *Fusarium* head blight and, additionally, they are able to produce mycotoxins in wheat grains [54,55,56,57]. Furthermore, less aggressive species, such as *Fusarium langsethiae* and *Fusarium poae*, are also able to produce mycotoxin in infected hosts [58,59]. A study on fungal community dynamics in a wheat-soybean rotation system showed that incorporating wheat residues into the soil significantly reduced plant pathogen associated fungal taxa including, Fusarium and Alternaria in the soil at the early phrase of decomposition (0–60 days) [2]. In contrast, our results showed not only wheat pathogens *Fusarium* spp. and *Alternaria* spp. but others were also detected in wheat residues over the same time span. This challenges the idea that wheat residue return can significantly reduce plant pathogens in the soil. Crop rotation could play an important role in determining the occurrence of plant pathogens in wheat fields [2].

Our results show that most of the plant pathogens in the ambient climate treatment are resilient to future climate conditions (except *Colletotrichum acerbum*, which is not active). Due to the relatively small (but realistic) changes in temperature and precipitation patterns implemented in the *GCEF* experiment, it is not surprising that actual endemic species do not disappear. The abundant fungal species in wheat residues *Zymoseptoria tritci*, *Microdochium nivale*, *Fusarium graminearum*, *F. tricinctum*, *F. poae*, *Gibellulopsis piscis*, *Mycosphaerella tassiana*, *Pseudopithomyces rosae,* and *Stemphylium vesicarium* form the backbone of the plant pathogenic mycobiome that can persist under the predicted climate change conditions. This is consistent with previous studies that have reported increased incidence and severity of head blight (caused by *Fusarium graminearum*) and *Septoria tritici* blotch (caused by *Zymoseptoria tritci*) on wheat exposed to predicted climate change due to elevated CO_2_ concentrations [60,61].

### 4.3. Future Climate Favors New Plant Pathogens Colonizing the Senescing Wheat Litter

Changes in rainfall patterns and temperature increases have already been found to favor the occurrence of novel plant pathogens [62]. Pathogen reproduction and survival are influenced by temperature [63]. Climate change parameters have also been described to support epiphytic and saprophytic stages of certain pathogens [21]. Thus, faced with abiotic changes, phytopathogens can change their lifestyle and trophic strategies to circumvent host defense strategies over a prolonged period. Our results indicate that the predicted climate changes, with alterations to temperature and precipitation patterns, can increase disease risks in agroecosystems, as under the future climate scenario applied in the *GCEF* field experiment, five potential plant pathogenic fungi (*Paraphoma rhaphiolepidis*, *Pyrenochaetopsis leptospora*, *Neostagonospora elegie*, *Blumeria graminis*, and *Waitea circinata*) emerged but were not detected in plots under the ambient climate conditions. Of these, *Blumeria graminis* has been reported to be a wheat powdery mildew pathogen [64]. *Rhizoctonia zeae*, the anamorph of *Waitea circinate*, is a causal agent of damping off of various wheat varieties [65]. To the best of our knowledge, this is the first report indicating that climate changes can affect the occurrence of fungal plant pathogens in wheat residue.

### 4.4. Why Do Fungal Community Dynamics Differ between Ambient and Future Climates? The Initial Mycobiome Colonizing Wheat Residues Determines Future Community Development

On the one hand, the composition of total, pathogenic, and saprotrophic fungal communities has changed under future climate treatment due to losses and gains; some members disappeared, while others emerged. This response is expected as a consequence of climate change but has never before been reported in the case of residue-borne wheat pathogens. On the other hand, under the ambient climate, we found a stable community over time. Previous studies have shown that community assembly history is one of the main determinants of microbial community structure and function [16,66]. In our experiment, climate manipulation started, in 2014, in a continuous realistic conventional farming situation. As a result, after four years, we found that the initial fungal communities colonizing wheat straw under the future climate treatment were already changed as compared with those under the ambient climate treatment. For instance, under the future climate at 0 days, 33% of the detected OTUs were specific. In addition, the relative abundance of some genera such as Fusarium increased significantly, while Musidium decreased. Later, after 30 and 60 days of field incorporation, strong succession patterns for the overall community, the saprotrophic and plant pathogenic fungi were detected only in case of future climate plots. We attributed this shift in the dynamics of the mycobiome to the initial community composition of the colonizing mycobiome, as the abundance of species that first colonize the wheat residues affect the succession of species that colonize the plant residues later according to the community assembly history hypothesis [16,66].

## 5. Conclusions

Our analyses showed a potentially high impact of climate change on fungal richness pattern and community dynamics in wheat residue at the early stage of decomposition. Surprisingly, we found fungal pathogens to be dominant over saprotrophs under both ambient and future climates, however, their richness pattern and community dynamics differed over time between the two climate treatments. Combining an NGS approach with the bromodeoxyuridine immunocapture technique demonstrated that these wheat residue pathogenic mycobiomes were mainly active. This indicates that straw incorporation into the soil can favor the contamination of wheat plants in the subsequent growing season. While pathogens of the current climate were resilient, additional ones occurred under the future climate scenario, which suggests an increased pathogen risks. This reinforces the need to monitor the richness and community dynamics of the pathogenic mycobiome associated with diverse crops under climate change conditions [27], and to be prepared for the possibility of increased risk of pathogens on crops as a result of climate change.

## Supplementary Materials

The following are available online at https://www.mdpi.com/2076-2607/8/6/908/s1, Figure S1: Schematic overview of the experimental design, Figure S2: Effects of climate manipulation on (A) total precipitation (sum of season) and (B) soil temperature (daily mean temperature), Figure S3: Venn diagram showing the overlap and distribution of fungal OTUs detected on wheat straw at 0 days under ambient and future climate treatments, Table S1: Relative abundance of phytopathogenic fungal species retrieved from OTUs colonizing wheat straw residues based on the UNITE species hypothesis along with the full name according to Index Fungorum.
