# Supplementary material for: Future Climate Significantly Alters Fungal Plant Pathogen Dynamics during the Early Phase of Wheat Litter Decomposition
Source: Microorganisms. 2020 Jun 16;8(6):908. doi: 10.3390/microorganisms8060908 (PMC7356542; doi:10.3390/microorganisms8060908)
Supplement: Supplementary file 1 [file microorganisms-08-00908-s001.zip › Supplementary_wheat_pathobiome/Supplementary_FigureS2.docx]

Supplementary materials

Article

Future climate significantly alters dynamics of fungal plant pathogens during the early phase of wheat litter decomposition

**Sara Fareed Mohamed Wahdan ^1,2,^*^,‡^, Shakhawat Hossen ^1,3,‡^ , Benjawan Tanunchai ^1^,
Martin Schädler ^4,5^, François Buscot ^1,5,†^ and Witoon Purahong ^1,^*^,†^**

^1^ Department of Soil Ecology, UFZ-Helmholtz Centre for Environmental Research, Theodor-Lieser-Str. 4, 06120 Halle (Saale), Germany; shakhawat.hossen@ufz.de (S.H.) tanunchai.benjawan@ufz.de (B.T.); francois.buscot@ufz.de (F.B.)

^2^ Department of Botany, Faculty of Science, Suez Canal University, 41522 Ismailia, Egypt

^3^ Friedrich-Schiller-Universität Jena, Institute of Ecology and Evolution, Dornburger Str. 159, 07743 Jena, Germany

^4^ UFZ-Helmholtz Centre for Environmental Research, Department of Community Ecology, Theodor-Lieser- Str. 4, 06120 Halle (Saale), Germany; martin.schaedler@ufz.de

^5^ German Centre for Integrative Biodiversity Research (iDiv) Halle-Jena-Leipzig, Deutscher Platz 5e, 04103 Leipzig, Germany

* Correspondence: sara-fareed-mohamed.wahdan@ufz.de, [sarah_wahdan@science.suez.edu.eg](mailto:sarah_wahdan@science.suez.edu.eg) (S.F.M.W.), witoon.purahong@ufz.de (W.P.); Tel.:+49 345 558 5207

^†^ Senior Authors.

^‡^ These authors contributed equally to this work.

**Figure S2**. Effects of climate manipulation on (**A**) total precipitation (sum of season) and (**B**) soil temperature (daily mean temperature) in a depth of 1 cm in experimental plots managed by conventional farming in the GCEF. Precipitation is not manipulated during the winter months. Note that the effects of soil temperature is strongly modulated by indirect effects via the change of vegetation cover (see also Schädler et al. 2019). Here, better growing conditions during the establishment of winter rape (autumn-spring) in the future treatment (higher precipitation, warmer) led to a higher vegetation cover and consequently lower direct insolation and lower soil temperatures in this treatment. This could not be observed for winter wheat in 2018 since plant growth was generally weak due to the generally low amount of precipitation.
