# Supplementary material for: Future Climate Significantly Alters Fungal Plant Pathogen Dynamics during the Early Phase of Wheat Litter Decomposition
Source: Microorganisms. 2020 Jun 16;8(6):908. doi: 10.3390/microorganisms8060908 (PMC7356542; doi:10.3390/microorganisms8060908)
Supplement: Supplementary file 1 [file microorganisms-08-00908-s001.zip › Supplementary_wheat_pathobiome/Supplementary_FigureS3.docx]

Supplementary materials

Article

Future climate significantly alters dynamics of fungal plant pathogens during the early phase of wheat litter decomposition

**Sara Fareed Mohamed Wahdan ^1,2,^*^,‡^, Shakhawat Hossen ^1,3,‡^ , Benjawan Tanunchai ^1^,
Martin Schädler ^4,5^, François Buscot ^1,5,†^ and Witoon Purahong ^1,^*^,†^**

^1^ Department of Soil Ecology, UFZ-Helmholtz Centre for Environmental Research, Theodor-Lieser-Str. 4, 06120 Halle (Saale), Germany; shakhawat.hossen@ufz.de (S.H.) tanunchai.benjawan@ufz.de (B.T.); francois.buscot@ufz.de (F.B.)

^2^ Department of Botany, Faculty of Science, Suez Canal University, 41522 Ismailia, Egypt

^3^ Friedrich-Schiller-Universität Jena, Institute of Ecology and Evolution, Dornburger Str. 159, 07743 Jena, Germany

^4^ UFZ-Helmholtz Centre for Environmental Research, Department of Community Ecology, Theodor-Lieser- Str. 4, 06120 Halle (Saale), Germany; martin.schaedler@ufz.de

^5^ German Centre for Integrative Biodiversity Research (iDiv) Halle-Jena-Leipzig, Deutscher Platz 5e, 04103 Leipzig, Germany

* Correspondence: sara-fareed-mohamed.wahdan@ufz.de, [sarah_wahdan@science.suez.edu.eg](mailto:sarah_wahdan@science.suez.edu.eg) (S.F.M.W.), witoon.purahong@ufz.de (W.P.); Tel.:+49 345 558 5207

^†^ Senior Authors.

^‡^ These authors contributed equally to this work.


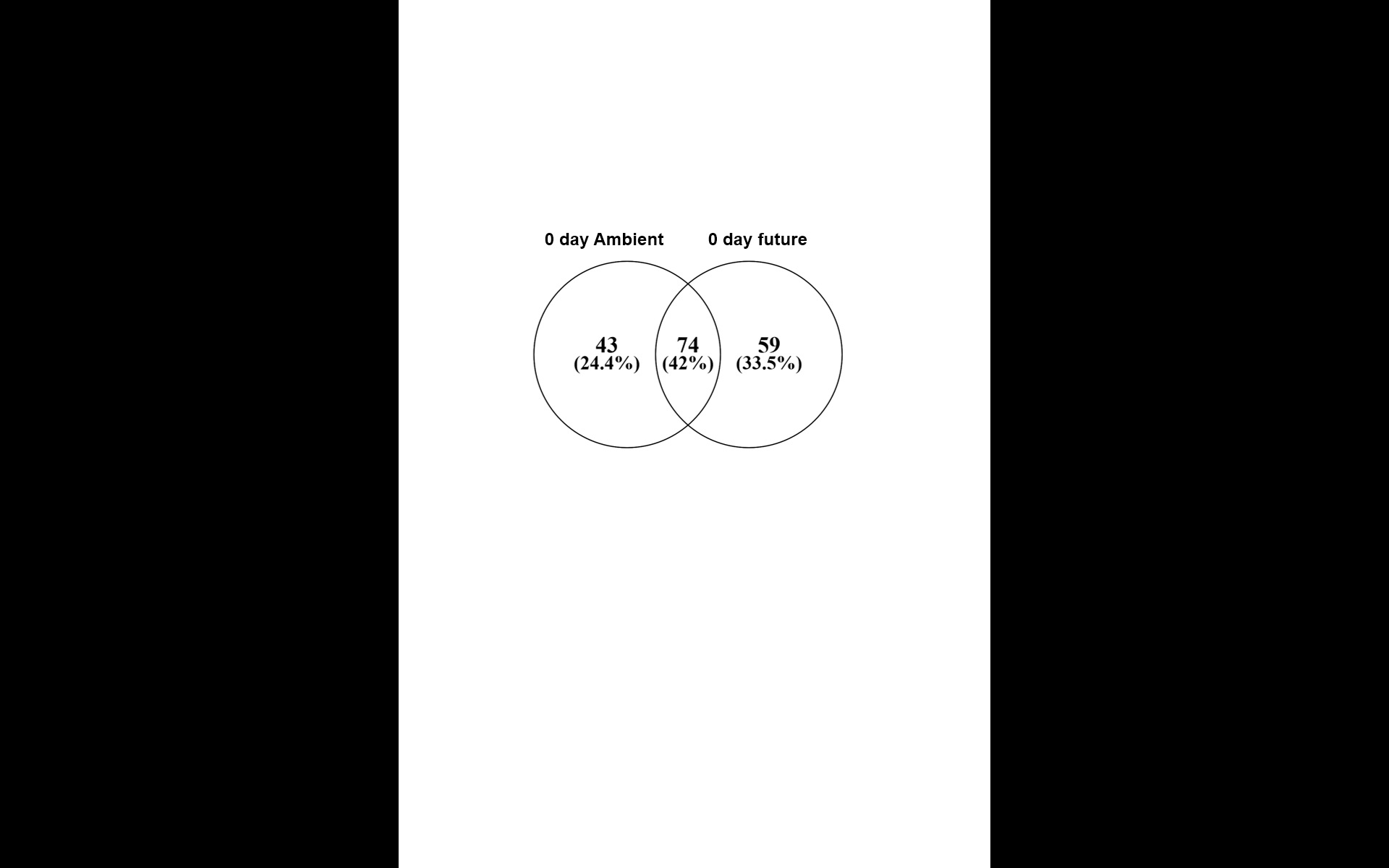


**Figure S3.** Venn diagram showing the overlap and distribution of fungal OTUs detected on wheat straw at 0 days under ambient and future climate treatments.
